# Improved quantification of muscle insulin sensitivity using oral glucose tolerance test data: the MISI Calculator

**DOI:** 10.1038/s41598-019-45858-w

**Published:** 2019-06-28

**Authors:** Shauna D. O’Donovan, Michael Lenz, Gijs H. Goossens, Carla J. H. van der Kallen, Simone J. M. P. Eussen, Coen D. A. Stehouwer, Marleen M. van Greevenbroek, Miranda T. Schram, Simone J. Sep, Ralf L. M. Peeters, Ellen E. Blaak, Natal A. W. van Riel, Theo M. C. M. de Kok, Ilja C. W. Arts

**Affiliations:** 1 Maastricht Centre for Systems Biology (MACSBIO), Maastricht University, Maastricht, The Netherlands; 2 Department of Human Biology, NUTRIM School of Nutrition and Translational Research in Metabolism, Maastricht University, Maastricht, The Netherlands; 3 Department of Internal Medicine, Maastricht University Medical Center+, Maastricht, The Netherlands; 4 Cardiovascular Research Institute Maastricht (CARIM), Maastricht University, Maastricht, The Netherlands; 5 Department of Epidemiology, Maastricht University, Maastricht, The Netherlands; 6 Heart and Vascular Center, Maastricht University Medical Center+, Maastricht, The Netherlands; 7 School for Public Health and Primary Care (CAPHRI), Maastricht University, Maastricht, The Netherlands; 8 Department of Rehabilitation Medicine, Maastricht University, Maastricht, The Netherlands; 9 Department of Data Science & Knowledge Engineering, Maastricht University, Maastricht, The Netherlands; 10 Department of Biomedical Engineering, Eindhoven University of Technology, Eindhoven, The Netherlands; 11 Department of Toxicogenomics, GROW School of Oncology and Developmental Biology, Maastricht University, Maastricht, The Netherlands

**Keywords:** Metabolic syndrome, Risk factors

## Abstract

The Muscle Insulin Sensitivity Index (MISI) has been developed to estimate muscle-specific insulin sensitivity based on oral glucose tolerance test (OGTT) data. To date, the score has been implemented with considerable variation in literature and initial positive evaluations were not reproduced in subsequent studies. In this study, we investigate the computation of MISI on oral OGTT data with differing sampling schedules and aim to standardise and improve its calculation. Seven time point OGTT data for 2631 individuals from the Maastricht Study and seven time point OGTT data combined with a hyperinsulinemic-euglycaemic clamp for 71 individuals from the PRESERVE Study were used to evaluate the performance of MISI. MISI was computed on subsets of OGTT data representing four and five time point sampling schedules to determine minimal requirements for accurate computation of the score. A modified MISI computed on cubic splines of the measured data, resulting in improved identification of glucose peak and nadir, was compared with the original method yielding an increased correlation (ρ = 0.576) with the clamp measurement of peripheral insulin sensitivity as compared to the original method (ρ = 0.513). Finally, a standalone MISI calculator was developed allowing for a standardised method of calculation using both the original and improved methods.

## Introduction

Insulin resistance is an important feature of the metabolic syndrome, which has been associated with approximately a threefold increased risk for the development of type 2 diabetes mellitus and cardiovascular disease^1^. Insulin resistance can occur in several tissues including the liver, skeletal muscle, and adipose tissue^2,3^. The severity of insulin resistance has also been found to vary between the tissues leading to a range of phenotypes^4–6^ including dyslipidemia^7^, disturbed glucose metabolism, and hyperinsulinemia^8^. Interventions, such as reduced caloric intake, have been shown to reduce whole-body insulin resistance^9^. However, several studies have demonstrated that certain interventions exert more or less tissue-specific effects, metformin administration improves hepatic insulin sensitivity^10^ and data suggests that increased physical activity predominantly affects skeletal muscle insulin sensitivity^11^. This tissue-specificity of interventions emphasises the importance of being able to accurately quantify tissue-specific insulin sensitivity. Currently, a hyperinsulinemic-euglycaemic clamp^12^ combined with a glucose tracer infusion^13,14^ is the gold standard for quantifying peripheral insulin resistance. Unfortunately, this method is costly, invasive, and time-consuming, making it difficult to implement in a clinical setting or on a large scale. These difficulties have been a motivating factor in the development of alternative methods.

Several metrics exist which attempt to quantify insulin sensitivity in the body as a whole using glucose and insulin measurements either after fasting (HOMA-IR^15^, QUICKI^16^) or during an Oral Glucose Tolerance Test (OGTT) (Matsuda^17^, Stumvoll^18^). These scores are widely used throughout metabolic and epidemiological research. In their 2007 paper, Abdul-Ghani *et al*. developed and validated tissue specific insulin sensitivity scores for muscle and liver^19^. The muscle insulin sensitivity index (MISI) has been validated through comparison with a hyperinsulinemic-euglycaemic clamp combined with a glucose tracer infusion (n = 155, Spearman correlation of ρ = 0.78, p < 0.0001).

The validity of the MISI score was subsequently questioned by several authors, reporting still significant, but considerably lower correlations of 0.542^20^, 0.55^21^, and 0.41^22^ with the hyperinsulinemic clamp. Moreover, Wilson and Ross^21^ also investigated the standard error of estimate of MISI, their results indicating a lack of precision with the score. While these lower correlations have been attributed to differences in the heterogeneity of study populations, particularly with regard to glucose tolerance status and BMI range^23,24^, the circumstances in which the application of the MISI score is appropriate and the factors contributing to such low correlation values in other studies still requires further evaluation.

Here, we investigated the dependence of MISI on the number of measurement time points during an OGTT, identifying minimal requirements for calculation in order to ensure an accurate estimation of MISI. Furthermore, we evaluated both the original and proposed modified MISI score with improved numerics (introduced in this article) based on a comparison to a hyperinsulinemic-euglycaemic clamp and describe numerical issues in the original MISI score calculations that contribute to the reported low correlations with the clamp.

In addition, as the original article^19^ did not specify measurement units or a precise method of calculation, the MISI score has been calculated with substantial variation in literature to date^25^. As a result MISI is most frequently used to rank individuals into relative muscle insulin sensitivity tertiles^26^, making comparison of MISI scores across studies impossible. Therefore, we have created a downloadable stand-alone MISI calculator for the automated and standardised calculation of MISI using both the original method and proposed modified method. The calculator also automatically flags OGTT curves for which the calculated MISI value may not be meaningful. This is of particular importance when automating the computation of MISI for large data sets which are increasingly becoming available.

## Research Design and Methods

### Standardising original MISI calculation

The MISI formula is based on the assumption that the decay of plasma glucose during an OGTT is primarily due to uptake by skeletal muscle, as endogenous production of glucose from liver will be inhibited by the post load increase in insulin^19^. It is calculated as

1 $$MISI=\frac{dG}{dt}/\bar{I}$$

where dG/dt denotes the slope of the regression line from the peak of the plasma glucose curve to its nadir and $\bar{I}$ is the average insulin concentration over the duration of the OGTT. There has been some variation in the implementation of MISI to date, most notably the omission of 0 minute time point when computing $\,\bar{I}$^25^. Hence, we propose a standardised original MISI were $dG/dt\,\,$is measured in µmol/L/min and computed as the line of best fit from the peak to the nadir, excluding any glucose rebound, and $\bar{I}$ is the mean measured insulin over the full 120 minutes in pmol/L.

### Modified MISI

The original MISI calculation is heavily dependent on the sampling times during an OGTT. Specifically, the calculation of $dG/dt\,\,$is critical since it relies on the identification of the maximum and minimum plasma glucose concentrations over the duration of the OGTT, which is currently limited to the discrete set of measured time points; with the true peak/nadir often occurring between two sampled time points. The computation of $\bar{I}$ (mean insulin concentration) is also sensitive to the sampling times, particularly in the case of unequal sampling intervals, which results in an increased weight of the more densely sampled intervals of the insulin curve. Such unequal sampling intervals are common with four (e.g. 0, 30, 60, 120 min) or seven (e.g. 0, 15, 30, 45, 60, 90, 120 min) time point OGTTs.

**Figure 1:**
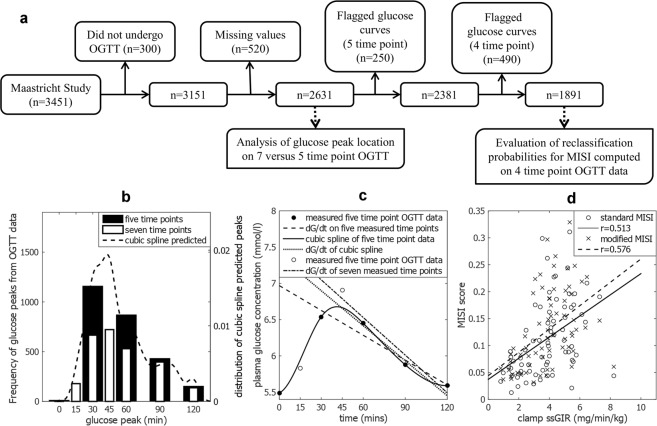
Overview of exclusion criteria for Maastricht Study data and results of analysis in PRESERVE and Maastricht studies. (**a**) Flow chart of exclusion of data from the Maastricht Study for analysis. (**b**) Bar plots of time of glucose peak in Maastricht study OGTT data, white bars show seven time point data; black bars indicate the five time point subset. Dashed line shows distribution of peak glucose time predicted by cubic splining of five time point data. (**c**) dG/dt five (dashed line) computed on a five time point subset (black circles) differs from dG/dt seven (dot-dashed line) computed on a seven time point OGTT (black circles plus empty circles). dG/dt spline (dotted line) computed on a cubic spline of the five time point data (solid line) more closely represents the seven time point dG/dt. (**d**) Scatter plot of PRESERVE Study (n = 69) clamp ssGIR with both the standard MISI (open circles) and cubic spline MISI (crosses). Correlation for standard MISI and modified MISI with ssGIR value are represented by the solid and dashed lines of best fit respectively.

In order to overcome these problems, we propose to interpolate the OGTT data with a cubic spline, inferring smooth glucose and insulin curves that allow for improved prediction of the peak glucose time and concentration while also accounting for unequal sampling intervals. Cubic splining is achieved by the piecewise fitting of third order polynomials between the measured data points such that the first and second derivatives at each measured point are continuous. In addition, we assume that the first and second derivatives at the start of the glucose curve (0 min) are zero, imposing a steady state condition. The regression line used to determine dG/dt is now fitted to the full inferred glucose curve rather than a limited number of sampled points (Fig. 1c), with a prediction of the true glucose peak and nadir. $\bar{I}$ is also computed on the full inferred insulin curve, thereby improving MISI calculation. Herein, this cubic spline method will be referred to as “modified MISI”.

### Flagging of problematic OGTT curves

**Figure 2:**
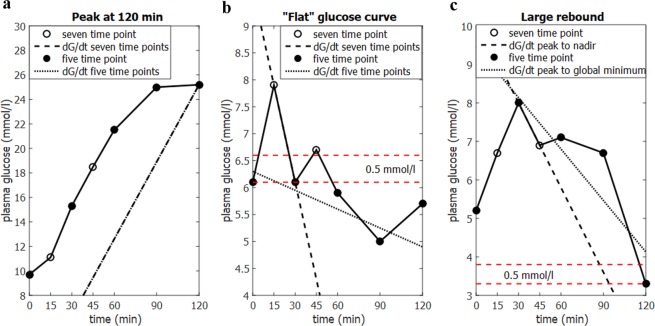
Criteria for flagging glucose curves when calculating MISI. (**a**) No unique solution for calculating dG/dt when the glucose peak occurs at 120 minutes. (**b**) Glucose curves with a glucose peak less than 0.5 mmol/l greater than the fasting value have often failed to capture the glucose peak and tend to yield low MISI values (dotted line), whereas the true value may be quite high (dashed line computed on seven time points). (**c**) Larger glucose rebound may not be negligible, strictly using the nadir may result in biologically irrelevant MISI scores.

Comparison of five and seven time point OGTT data from the Maastricht Study^27^ identified several occasions where it is either not possible to calculate MISI or where, given the number of time points, the calculated MISI value might not be biologically meaningful. The criteria for flagging OGTT curves are as follows (Fig. 2):

1. The glucose curve has a peak at 120 min (no unique solution for dG/dt)
2. The glucose curve appears to be flat, defined as peak glucose concentration (G_peak_) being less than 0.5 mmol/L greater than the fasting value (G_0_): G_peak_ < G_0_ + 0.5 mmol/L
3. The glucose curve has a non-negligible rebound, i.e. the nadir is not the global minimum for the glucose curve over 120 minutes. Defined as having a glucose rebound more than 0.5 mmol/L greater than global minimum.

### Datasets and measurements

In order to examine the effect of different sampling schedules and population composition on the computation of MISI and modified MISI we use data from the Maastricht Study^27^ and PRESERVE Study^28^.

### The Maastricht study

The Maastricht Study is an observational prospective population-based cohort study. The rationale and methodology have been described previously^27^. In brief, the study focuses on the etiology, pathophysiology, complications, and comorbidities of type 2 diabetes and is characterised by an extensive phenotyping approach. All individuals aged between 40 and 75 years and living in the southern part of the Netherlands were eligible for participation. Participants were recruited through mass media campaigns and from the municipal registries and the regional Diabetes Patient Registry via mailings. Recruitment was stratified according to known type 2 diabetes status, with an oversampling of individuals with type 2 diabetes, for reasons of efficiency. The present report includes cross-sectional data from the first 3451 participants, who completed the baseline survey between November 2010 and September 2013. The examinations of each participant were performed within a time window of three months. The study has been approved by the institutional medical ethical committee (NL31329.068.10) and the Minister of Health, Welfare and Sports of the Netherlands (Permit 131088-105234-PG). All participants gave written informed consent. Provided participants were not using exogenous insulin and had a fasting plasma less than 11 mmol/L they underwent a seven time point 75 g OGTT (0, 15, 30, 45, 60, 90, 120 mins). Removing individuals with missing time points, complete seven time point OGTT data was available for 2631 individuals, (Fig. 1a), 1584 with normal glucose tolerance, 124 with impaired fasting glucose, 305 with impaired glucose tolerance, and 616 with type 2 diabetes, cut-offs defined by WHO 2006 criteria^29^. Subjects had a wide range of BMI (14.38–52.25 kg/m^2^) [mean 26.81 ± standard deviation 4.32], a mean age of 59.7 ± 8.2 years and were 52.1% male (Supplemental Table S1). For subsequent analysis all flagged glucose curves were excluded, with OGTT data for 1891 individuals used to compare common OGTT sampling schedules.

### PRESERVE study

The PRESERVE Study is a randomised controlled, double-blind, two-centre study in which 79 individuals with impaired fasting glucose and/or impaired glucose tolerance were randomized to receive 320 mg of valsartan or placebo for 26 weeks in order to evaluate the effects of the angiotensin II type 1receptor blocker on insulin sensitivity and β-cell function^28^ (Clinical trial reg. no. ISRCTN42786336). The study was conducted in accordance with the Declaration of Helsinki and was approved by the local ethics committee. All participants gave written informed consent. At baseline, all individuals underwent a seven time point 75 g OGTT (t0, 10, 20, 30, 60, 90, 120 mins) and a hyperinsulinemic-euglycaemic clamp with an insulin infusion rate of 40 mU/min/m^2^. The steady state glucose infusion rate over the final 30 min of the hyperinsulinemic-euglycaemic clamp (ssGIR, mg * min^−1^ * kg^−1^) was used to calculate peripheral insulin sensitivity.

Data for 71 out of the 79 individuals was used, excluding five individuals with missing values in OGTT or clamp data, and three individuals for whom MISI could not be computed as their peak glucose concentration occurred at 120 minutes. Of the remaining 71 individuals (45% male, average age of 58.2 ± 6.9 years, mean BMI of 29.8 ± 4.5 kg/m^2^) 39 had impaired fasting glucose, 11 had impaired glucose tolerance, and 21 had both impaired fasting glucose and impaired glucose tolerance, using glucose metabolism cut-offs as defined by WHO 2006^29^. Two further individuals were classed as statistical outliers for MISI due to acute hypoglycaemia at 90 minutes (<2.5 mmol/L), resulting in very high MISI values. Results are provided both including and excluding these individuals (Supplemental Table S2).

### The MISI calculator

**Figure 3:**
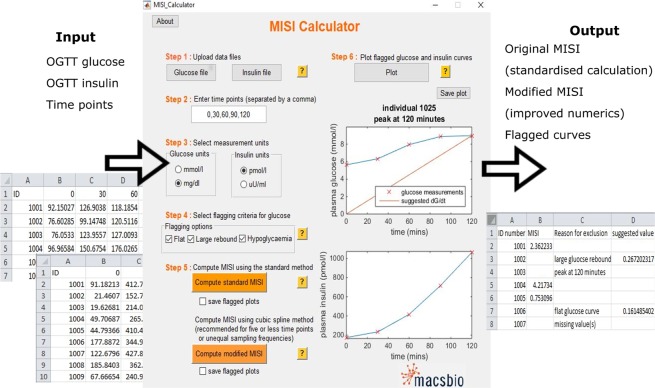
MISI score calculator indicating input and output for its use. The MISI Calculator take time series of glucose and insulin measurements collected during and OGTT for multiple individuals stored in the form of Excel files as input. The calculator can convert measurement units of both glucose and insulin to SI units used for calculation. The user can the specify flagging criteria and method for the calculation of MISI. The user can choose to save plots of the glucose and insulin curves when calculating the MISI score or view the flagged glucose and insulin curves in the MISI Calculator app. Output of the calculate is an excel file containing the user supplied ID number and the calculated MISI value. If MISI was not calculated, either due to flagging or missing values, an explanation is provided along with a suggested MISI value where possible.

The MISI calculator was programmed in MATLAB 2014b, The MathWorks Inc., Natick, Massachusetts, United States of America. and the stand alone graphical user interface (GUI) was generated to install Matlab Runtime, such that no Matlab installation is required for the use of the calculator (Fig. 3). Both the source code and the stand-alone calculator can be downloaded from www.maastrichtuniversity.nl/misi. A tutorial and example data files are also provided.

### Calculations and statistics

In order to evaluate the performance of both the standard and modified MISI the following analyses were performed using each method and compared.

In order to test the effect of sampling frequency on performance MISI calculated on subsets of data representing several standard OGTT sampling schedules derived from the Maastricht Study OGTT data (n = 1891) were compared. Seven time points (t0, 15, 30, 45, 60, 90, 120 min), five time points (t0, 30, 60, 90, 120 min) and several four time point subsets omitting various time points from the five time point subset were compared. Spearman correlations with standard five time point sampling schedule with permutation based p-values and reclassification probabilities were computed, as this was the sampling frequency validated in the original article^19^. Reclassification probabilities between two scores were calculated as the number of samples that were classified into two different insulin resistance tertiles by each score, divided by the total number of samples. This approach was repeated within the PRESERVE Study data set (n = 71), comparing the MISI computed using both methods on five time point and the various four time point subsets to the ssGIR of the hyperinsulinemic-euglycaemic clamp.

It has been reported that the heterogeneity of the population being examined may have an impact on the strength of the correlation between MISI and the clamp-based insulin sensitivity^20,23^ with a wider spread in values yielding a higher correlation^24^. To further investigate this relationship and to test the robustness of our correlation results, a bootstrapping-like approach was used, in which 1000 subsets of various sizes (50–70 individuals) were randomly drawn from the 71 individuals of the PRESERVE dataset and evaluated based on their Spearman correlation between both the standard and modified MISI and the clamp-based insulin sensitivity.

## Results

### Modified MISI

Analysis of the Maastricht Study seven time point OGTT demonstrated that 27% of the total population studied (722 of 2631 individuals) had a peak glucose concentration at 45 mins (Fig. 1b, white bars). Had the OGTT been sampled at just five time points (t0, 30, 60, 90, 120 min) (Fig. 1b, black bars), the majority of individuals with a peak glucose at 45 min would be distributed between having a glucose peak at 30 min (366 of 722 individuals) or 60 min (324 of 722 individuals). With some individuals now having a peak at 90 min or later (32 of 722), introducing potential inaccuracy in the resulting MISI values as the true concentration of the glucose peak will be underestimated. Cubic splining of this five time point OGTT subset allowed for the peak glucose concentration to be inferred. Out of the 722 individuals with a peak at 45 minutes in the seven time point data, 409 were assigned a peak glucose concentration between 37.5 mins and 52.5 mins, enabling the recovery of information which may potentially have been lost as a result of less frequent sampling.

A five time point subset was generated from the PRESERVE Study OGTT data using 0, 30, 60, 90, and 120 min time points to reflect a standard OGTT. The clamp ssGIR was compared to both the standard and modified MISI computed on the five time point subset using Spearman correlation. The modified method yielded a non-significant increase in correlation with ssGIR over the standard method ρ = 0.576 versus ρ = 0.513 excluding the two statistical outliers (n = 69). Retaining the two outliers the same trend is observed with the modified MISI yielding a correlation of ρ = 0.526 with the ssGIR versus ρ = 0.469 for the standard method (n = 71). (p < 0.001 for all correlation coefficients).

### MISI applied to OGTT with varying temporal information

In order to determine the minimum requirements for calculation, both the standard and modified MISI were computed on various four time point subsets of OGTT data and compared with the ssGIR of the hyperinsulinemic euglycaemic clamp for 66 individuals in the PRESERVE study. Results, shown in Table 1, indicate poor correlation between standard MISI computed on four time points and the clamp ssGIR, particularly when the 30 or 60 minute time point was absent (ρ = 0.440 and ρ = 0.356 respectively). The modified MISI calculated on four time points yields a higher correlation with the clamp ssGIR than the standard MISI. When the 60 minute time point was missing the correlation coefficient increases from ρ = 0.356 for the standard MISI to ρ = 0.618 for the modified MISI.

**Table 1:** Spearman correlations and reclassification probabilities for MISI computed using both the standard and modified methods on OGTT data for various common sampling schedules.

Correlation	Maastricht Study (n = 1891)				PRESERVE Study (n = 66)			
	5 time point MISI		7 time point MISI		5 time point MISI		clamp ssGIR	
time points	standard	modified	standard	modified	standard	modified	standard	modified
4 (30, 60, 90, 120)	0.999	0.945	0.924	0.819	0.999	0.944	0.473	0.457
4 (0, 60, 90, 120)	0.928	0.881	0.878	0.794	0.897	0.826	0.440	0.464
4 (0, 30, 90, 120)	0.817	0.842	0.775	0.763	0.831	0.852	0.356	0.618
4 (0, 30, 60, 120)	0.790	0.703	0.748	0.664	0.886	0.755	0.481	0.661
5 (0, 30, 60, 90, 120)	1	1	0.925	0.864	1	1	0.475	0.561
**Reclassification**	**5 time point MISI**		**7 time point MISI**		**5 time points MISI**		**clamp ssGIR**	
**time points**	**standard**	**modified**	**standard**	**modified**	**standard**	**modified**	**standard**	**modified**
4 (30, 60, 90, 120)	0.015	0.202	0.151	0.277	0.030	0.152	0.470	0.576
4 (0, 60, 90, 120)	0.160	0.216	0.202	0.302	0.106	0.212	0.515	0.485
4 (0, 30, 90, 120)	0.326	0.292	0.348	0.347	0.349	0.227	0.500	0.485
4 (0, 30, 60, 120)	0.331	0.406	0.365	0.412	0.167	0.333	0.591	0.394
5 (0, 30, 60, 90, 120)	0	0	0.145	0.220	0	0	0.485	0.485

First two columns show comparison with MISI computed on five and seven time points within the Maastricht Study (n = 1891). The second two columns show comparison with MISI computed on five time points and the steady state glucose infusion rate of the hyperinsulinemic-euglycaemic clamp (ssGIR) for individuals within the PRESERVE Study. The upper half of the table shows correlation coefficients computed by Spearman correlation, the lower section shows net reclassification probabilities.

Within epidemiological and intervention studies to date MISI has typically been used to rank individuals into muscle insulin sensitivity tertiles^26,30^. Individuals within the Maastricht Study (n = 1891) were ranked into muscle insulin sensitivity tertiles using the standard MISI computed on the four time point subsets of data and compared with classification defined using the five time point data. Again, provided the 120 min time point was included the reclassification probability was less than 33%. Reclassification probability of MISI computed on seven time point data with MISI computed on five time point OGTT data was 15.2%. Looking more closely at the effect of reclassification of the composition of the muscle insulin sensitivity tertiles defined by both standard and modified MISI in the Maastricht Study data set (n = 1891) (Supplementary Table 3). It is of note that the least insulin sensitive group as defined by modified MISI has an increased mean whole body insulin resistance (as defined by HOMA) and contains a lower proportion of normal glucose tolerance (NGT) individuals than the group defined using standard MISI (55.2% for modified MISI versus 56.7% for standard MISI calculated on seven time points). Moreover, the reverse can be seen in the most insulin sensitive groupings; with the modified MISI group having a lower mean HOMA value and a greater number of NGT individuals than the group defined using standard MISI (65.1% for modified MISI versus 60.8% for standard MISI calculated on seven time points). These differences are statistically non-significant (using a two tailed t-test).

### Effect of population on correlation with clamp

**Figure 4:**
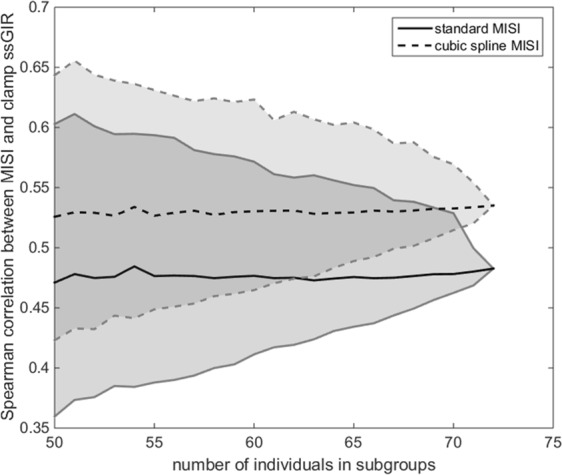
Spearman correlation values between standard and modified MISI and clamp ssGI for randomly selected sub-populations of PRESERVE study. Median spearman correlation between clamp ssGIR and both standard MISI (solid line) and cubic spline MISI (dashed line) for 1000 randomly selected subgroups of varying size from the PRESERVE Study. 95% quantiles are indicated by the shaded region to either side of the medians.

Several studies have reported much lower correlations between MISI and the clamp ssGIR than observed in the original paper^20–22^. These lower values have largely been attributed to the reduced heterogeneity of the populations being used, with a narrower range of muscle insulin sensitivity when compared to the original study^23,24^. Testing the sensitivity of the correlation between the MISI and the clamp ssGIR to population composition, we randomly selected 1000 sub-populations of increasing size from the PRESERVE study and compared the Spearman correlations achieved between MISI and the clamp ssGIR (Fig. 4). Looking at the range of the 95% quantiles, correlations of 0.36 to 0.60 with a median of 0.47 were possible when randomly selecting sub-populations of 50 individuals from the data set of 71 individuals using the standard method, demonstrating the variability of the Spearman correlation between MISI and the clamp. For the modified method there was an improved median correlation of 0.53 for the same sub-populations with 95% quantiles of 0.42 to 0.64.

### Flagged glucose curves

The original article already mentions that MISI is not appropriate for individuals with type 2 diabetes due to the peak occurring at 120 minutes^19^. There were 154 individuals (6% of total data set) with a peak at 120 minutes within the Maastricht Study, of whom 107 (69.5%) had type 2 diabetes. As there is no unique solution for $dG/dt\,$for a glucose curve with a peak at 120 minutes (Fig. 2a) it is not possible to compute MISI. However, it would be expected that these individuals would fall into the least muscle insulin sensitive tertile.

In addition, there were 47 individuals (2%) where the peak glucose concentration was less than 0.5 mmol/L greater than the fasting value (Fig. 2b). These curves often appeared quite flat when using a standard five time point sampling frequency and were assigned low MISI values, indicating low muscle insulin sensitivity. However, on examining the seven time point data most of these individuals would be classified among the most insulin sensitive, as their glucose peak occurred at 15 minutes, and glucose levels had returned to near fasting concentration by 30 minutes. These individuals tended to have lower BMI than the mean for the whole data set (24.1 ± 3.1 kg/m^2^) with 45 of the 47 individuals being classed as normal glucose tolerant (Supplemental Table S1).

Conversely, there were 58 individuals (2%) with large glucose rebounds. Abdul-Ghani *et al*.’s original article^19^ specifies that any glucose rebound should be excluded in the calculation of $dG/dt$, leading to some issues when automating the computation of MISI. This especially becomes problematic with more frequently sampled glucose curves, where fluctuations in the glucose curves as a result of measurement noise may introduce erroneous local minima (Fig. 2c). In order to compensate for this, our calculator excludes the rebound if it is less than 0.5 mmol/L greater than the minimum. If the rebound is larger than 0.5 mmol/L the glucose curve will be flagged; allowing the user to choose to accept the calculated value following visual inspection of the glucose curve.

### MISI calculator

The MISI calculator allows for the automated calculation of MISI in a standardised way. It consists of a graphical user interface which guides the user through uploading glucose and insulin OGTT data and specification of measurement time points (Fig. 3). It automatically filters for missing values and converts glucose and insulin measurements to SI units. The calculator allows the user to specify which criteria they wish to flag glucose curves for manual inspection and computes MISI using the standard or modified methods. The output file contains either the calculated MISI values, or an explanation as to why the calculation of MISI is not meaningful and, where possible, a suggested MISI value for each individual. The calculator also allows the user to visually inspect the flagged glucose curves and suggested MISI values.

## Discussion

Insulin resistance is often present in multiple tissues long before the onset of overt clinical disorders such as type 2 diabetes mellitus and cardio-vascular diseases. Abdul-Ghani *et al*.^19^ proposed a score to quantify muscle-specific insulin sensitivity using five time point OGTT data (t0, 30, 60, 90, 120 min) which was validated against a hyperinsulinemic-euglycaemic clamp combined with a glucose tracer infusion.

Several studies have failed to reproduce the correlation between MISI and the clamp reported in the original study^20–22^; these lower correlations are typically attributed to a reduced heterogeneity in the population composition. Analysis of correlations obtained for MISI computed on randomly selected sub-populations from the PRESERVE study, a relatively homogenous population with respect to BMI and glucose tolerance status when compared to the original study population^19^, with the clamp ssGIR highlighted the range of correlation coefficients which could be obtained through small variations in the population composition. Correlation for the standard MISI computed on five time point OGTT data with the clamp ssGIR for the whole data was 0.513, which is considerably lower than the 0.79 reported in the original study, but is comparable with values achieved by other studies^20–22^. Our proposed modified MISI method yields an improved correlation with the clamp of 0.576. While the increase in correlation is non-significant, the modified method is also numerically more robust than the original method. Application of the original index to a standard five time point OGTT would be expected to under-estimate the glucose peak and nadir, as demonstrated in Fig. 1b. The use of cubic splining in our modified MISI method allows for inference of the glucose peak and nadir while also correcting for unequal sampling intervals when calculating the mean insulin concentration. Spearman rank based correlation was used as the relationship between clamp ssGIR and MISI was observed to be non-linear. While the hyperinsulinemic-euglycaemic clamp performed during the PRESERVE study was not combined with a glucose tracer infusion, as the insulin infusion rate was the same as in Abdul-Ghani’s original paper (40 mU/min/m^2^)^19^ we assumed this is sufficient to fully suppress endogenous glucose production. We also acknowledge the need to further validate our modified MISI method, as well as the original index, on a range of heterogeneous populations. The composition of the PRESERVE study population, consisting only of individuals with impaired fasting glucose and/or impaired glucose tolerance, may limit the generalisability of our results.

Comparison of MISI computed on five versus seven time point OGTT data highlighted several, previously unreported, situations in which the numerical value obtained when automating the calculation of MISI may not be biologically relevant. Abdul-Ghani addressed issues with glucose curves with a peak at 120, advising the score not be applied to individuals with type 2 diabetes mellitus^19^. Our analysis also revealed several additional, potentially problematic, curves requiring manual inspection; namely ‘flat’ glucose curves, which may arise from a failure to capture a rapid response to the oral glucose bolus due to infrequent sampling, and curves, where due to erroneous identification of the nadir, the glucose rebound may not be negligible. While more frequent sampling should improve the accuracy of MISI as it will result in improved detection of the glucose peak. However, an increased number of time points also increases the chances of detecting a premature nadir. Our calculator flags glucose curves which display potentially problematic traits such as large rebounds or modest peaks, allowing for manual inspection of the MISI value. This is particularly useful when applying MISI to large data sets, as in the case of the Maastricht study, where manual inspection of each glucose curve is prohibitive due to population size.

As part of our analyses we also evaluated the use of the oral glucose minimal model to infer the full glucose curve from measured insulin data (work not shown). The oral glucose minimal model^31^ is a physiology based mathematical model which describes both insulin dependent and independent glucose dynamics following oral administration of a glucose load. Fitting of the oral glucose minimal model to the discretely sampled OGTT glucose data for each individual, using the measured insulin data as input, allowed for inference of the full glucose curve; including a prediction of the true glucose peak and nadir, as with cubic splining. However, as it was necessary to estimate a unique model parameter set for each individual in order to infer the glucose curve, this method is computationally much more heavy than cubic splining. Given the longer computation time necessary for use of the oral glucose minimal model, which becomes prohibitive for use in larger cohort studies, it was decided to use the cubic spline method.

As the authors of the original paper^19^ did not provide a detailed method for the calculation of MISI there has been considerable variation in the implementation of MISI in research to date^25,32^. As no set measurement units are specified, the MISI values reported in literature differ by several orders of magnitude^32–35^. Ergo, there is currently no cut-off as to what MISI value can be classed as muscle insulin resistance. Currently, MISI is most often used to rank individuals into muscle insulin sensitivity tertiles. Consequently, classification of muscle insulin resistance is relative to each data set making comparison of results across studies difficult. Variation in the computational method can also be seen in the literature, with some authors choosing to remove certain time points when computing MISI. Here, we have demonstrated that the exclusion of time points can have an impact on the resulting ranking akin to the reclassification probability for the four time point subsets reported in Table 1. Spearman correlations and reclassification probabilities for each of the four time point subsets with the ssGIR of the clamp in the PRESERVE study indicate that caution should be used when applying the standard MISI to four time point OGTT sampling schedules, in particular when the 30 or 60 minute time point is absent. The higher correlation coefficients obtained between the modified MISI method and the clamp ssGIR suggests the use of cubic splining offers the modified MISI method a robustness to the limited data and unequal sampling schedules of the four time point subsets of OGTT data. Based on these results we would advise using the modified method when computing MISI on four or five time point OGTT data, and not computing MISI using either method if the 120 min time point is not available.

In order to prevent these issues and standardise the computation of MISI we have developed the MISI Calculator. This calculator provides a user friendly tool, allowing the user to compute MISI using the standard method or the modified method, which is recommended when using data with limited temporal information or with unequally spaced sampling intervals, while also standardising both the computational method and units used. In the long term, we envision this standardised computation of MISI will allow for easier comparison of MISI across studies and the identification of a cut-off value of MISI indicative of clinical muscle insulin resistance.

## Supplementary information

Supplementary material

## Data Availability

Both Maastricht Study and PRESERVE Study data are unsuitable for public deposition due to ethical restriction and privacy of participant data. Data are available from The Maastricht Study and PRESERVE Study for any interested researcher who meets the criteria for access to confidential data. The Maastricht Study Management Team (research.dms@mumc.nl) may be contacted to request Maastricht Study data. Prof. Ellen Blaak (e.blaak@maastrichtuniversity.nl) may be contacted to request PRESERVE Study data.
