# Supplementary material for: Improved quantification of muscle insulin sensitivity using oral glucose tolerance test data: the MISI Calculator
Source: Sci Rep. 2019 Jun 28;9:9388. doi: 10.1038/s41598-019-45858-w (PMC6598992; doi:10.1038/s41598-019-45858-w)
Supplement: Supplementary file 1 — Supplementary material [file 41598_2019_45858_MOESM1_ESM.docx]

**Improved quantification of muscle insulin sensitivity using oral glucose tolerance test data: the MISI Calculator.**

Shauna D. O’Donovan^*^, Michael Lenz, Gijs H. Goossens, Carla J.H. van der Kallen, Simone J.M.P. Eussen, Coen D.A. Stehouwer, Marleen M van Greevenbroek, Miranda T. Schram, Simone J. Sep, Ralf L.M. Peeters, Ellen E. Blaak, Natal A.W. van Riel, Theo M.C.M. de Kok, Ilja C.W. Arts.

*shauna.odonovan@maastrichtuniversity.nl

**Supplementary Table 1: Demographics of relevant subsets of individuals from the Maastricht Study used in this study.**

|  | Maastricht Study data set | | Maastricht Study (full seven time point OGTT) | | Maastricht Study (removed curves flagged at 4 time points) | | Flat glucose curves (5 time point OGTT) | | Peak at 120 minutes (5 time points OGTT) | | Large glucose rebound (5 time point OGTT) | | |
| --- | --- | --- | --- | --- | --- | --- | --- | --- | --- | --- | --- | --- | --- |
| Number of individuals | 3451 | | 2631 | | 1891 | | 47 | | 154 | | 58 | |  |
| Age (years) | 59.8 ± 8.3 (40 – 76) | | 59.7 ± 8.2 (40 – 76) | | 60.1 ± 8.1 (40 – 76) | | 54.2 ± 8.4 (40 – 73) | | 62.5 ± 7.8 (41 – 75) | | 57.5 ± 7.9 (41 – 74) | |  |
| Sex (n, % male) | 1775 | 50.1 % | 1373 | 52.2% | 1066 | 56.4% | 9 | 19.2% | 69 | 44.8% | 17 | 29.3% |  |
| BMI (kg/m^2^) | 27.1 ± 4.6 (14.4 – 52.3) | | 26.8 ± 4.3 (14.4 – 52.3) | | 27.1 ± 4.3 (14.4 – 52.3) | | 24.2 ± 3.1 (18.2 – 31.3) | | 27.4 ± 4. 6 (17.9-41.5) | | 25.3 ± 3.8 (17.9 – 36.1) | |  |
| Normal glucose tolerance | 1924 | 55.8% | 1584 | 60.2% | 1164 | 61.6% | 45 | 95.7% | 27 | 17.5% | 54 | 93.1% |  |
| Impaired fasting glucose | 143 | 4.0% | 124 | 4.7% | 105 | 5.6% | 1 | 2.1% | 0 | 0.0% | 0 | 0.0% |  |
| Impaired glucose tolerance | 368 | 10.7% | 305 | 11.6% | 257 | 13.6% | 0 | 0.0% | 20 | 13.0% | 3 | 5.2% |  |
| Type 2 diabetes | 975 | 28.3% | 616 | 23.4% | 365 | 19.3% | 1 | 2.1% | 107 | 69.5% | 1 | 1.7% |  |
| Type 1 diabetes | 37 | 1.1% | - | - | - | - | - | - | - | - | - | - |  |
| Other type diabetes | 4 | 0.1% | - | - | - | - | - | - | - | - | - | - |  |
| Oral glucose lowering medication | 716 | 20.8% | 446 | 16.9% | 254 | 13.4% | 0 | 0.0% | 89 | 57.8% | 1 | 1.7% |  |
| Insulin use | 257 | 7.5% | - | - | - | - | - | - | - | - | - | - |  |

For Age and BMI both the mean ± the standard deviation are shown along with the range of values from minimum to maximum. For other variables the number of relevant individuals and the percentage of total population are supplied. Column one: full data set of 3451 participants, who completed the baseline survey between November 2010 and September 2013. Colums two: removing individuals who either did not undergo an OGTT, due to use of exogenous insulin or fasting glucose ≥11 mmol/L (n=300), or had one or more missing values for glucose or insulin during the OGTT (n=520) full seven time point OGTT data is available for 2631 individuals. This subset of individual is used to evaluate the location of the glucose peak in five versus seven time point OGTT data. Column three: all individuals with glucose curves which were flagged on four time point data were removed resulting in a final data set of 1891 individuals used for comparing reclassification probabilities with MISI ranking derived on five and seven time point OGTT data. Columns four through six: subsets of individuals whose glucose curves were flagged using five time point data.

**Supplementary Table 2: Demographics of participants from the PRESERVE Study**

|  | Preserve Study (including outliers) | | Preserve Study (excluding outliers) | |
| --- | --- | --- | --- | --- |
| Number of individuals | 71 | | 69 | |
| Age (years) | 58.3 ± 6.9  (38 – 69) | | 58.3 ± 6.9  (38-69) | |
| Sex (% male) | 54.9 % | | 55.1% | |
| BMI (kg/m^2^) | 29.8 ± 4.5  (22.7 – 44.39) | | 29.8 ± 4.5  (22.7 – 44.39) | |
| Impaired fasting glucose (IFG) | 39 | 54.9% | 37 | 53.6% |
| Impaired glucose tolerance (IGT) | 11 | 15.5% | 11 | 15.9% |
| Both (IFG & IGT) | 21 | 29.6% | 21 | 30.4% |

Demographics for participants used from the PRESERVE Study, both including and excluding two MISI score outliers. Age and BMI are given in mean ± standard deviation. Remaining variables indicate the glucose tolerance status of the participants described by the number of individuals and percentage of total population.

**Supplementary Table 3: Demographics of highest and lowest muscle insulin sensitivity tertile defined by both standard and modified MISI.**

| Reclassification of muscle insulin sensitivity tertiles (the Maastricht Study n=1891) | | | | |
| --- | --- | --- | --- | --- |
|  | Lowest muscle insulin sensitivity | | | |
|  | Five time points | | Seven time points | |
|  | Original MISI | Modified MISI | Original MISI | Modified MISI |
| BMI | 28.4 ± 4.6 | 28.5 ± 4.6 | 28.5 ± 4.7 | 28.6 ± 4.5 |
| HOMA | 4.11 ± 3.22 | 4.21 ± 3.24 | 4.18 ± 3.23 | 4.25 ± 3.23 |
| AGE | 60.6 ± 8.1 | 60.6 ± 8.0 | 60.4 ± 8.2 | 60.6 ± 8.1 |
| SEX (% female) | 52.1% | 56.0% | 53.7% | 55.7% |
| # NGT | 361 (57.3%) | 348 (55.2%) | 357 (56.7%) | 348 (55.2%) |
| # IFG | 30 (4.8%) | 32 (5.1%) | 29 (4.6%) | 32 (5.1%) |
| # IGT | 140 (22.2%) | 141 (22.4%) | 144 (22.9%) | 136 (21.6%) |
| #T2DM | 99 (15.7%) | 109 (17.3%) | 100 (15.9%) | 114 (18.1%) |
|  | Highest muscle insulin sensitivity | | | |
|  | Five time points | | Seven time points | |
|  | Original MISI | Modified MISI | Original MISI | Modified MISI |
| BMI | 26.0 ± 3.9 | 25.8 ± 3.8 | 26.0 ± 3.9 | 25.7 ± 3.7 |
| HOMA | 2.21 ± 1.56 | 2.13 ± 1.45 | 2.21 ± 1.61 | 2.13 ±1.52 |
| AGE | 59.9 ± 8.1 | 59.9 ± 8.0 | 59.9 ± 8.0 | 59.9 ± 7.9 |
| SEX (% male) | 39.1% | 40.0% | 39.9% | 41.4% |
| # NGT | 389 (61.8%) | 402 (63.8%) | 383 (60.8%) | 410 (65.1%) |
| # IFG | 44 (7.0%) | 41 (6.5%) | 40 (6.4%) | 42 (6.7%) |
| # IGT | 32 (5.1%) | 31 (4.9%) | 40 (6.4%) | 29 (4.6%) |
| #T2DM | 166 (26.4%) | 157 (24.9%) | 168 (26.7%) | 150 (23.8%) |

Demographics of highest and lowest muscle insulin sensitivity tertiles defined by both standard MISI and modified MISI calculated on five time point (0,30,60,90,120 minute) and seven time point (0,15,30,45,60,90,120) OGTT data from the Maastricht Study. BMI, HOMA, and AGE are given as the mean value for the tertile ± the standard deviation. Sex is given as percentage male. Glucose tolerance status is indicated as the number of individual of the glucose tolerance status and percentage of the full grouping. Glucose tolerance statuses are as follows; normal glucose tolerance (NGT), impaired fasting glucose (IFG), impaired glucose tolerance (IGT), and type 2 diabetes mellitus (T2DM). Each tertile consists of 630 individuals.
